# Urethral closure occurs by recoiling, pressure transmission, and a guarding reflex

**DOI:** 10.1007/s00192-020-04475-2

**Published:** 2020-09-16

**Authors:** Bo S. Bergström

**Affiliations:** Present Address: Karlavägen 27A, 11431, Stockholm, Sweden

**Keywords:** Mobility, TVT, Pascal's law of fluid pressures, Pathophysiology, Stress urinary incontinence, Urethral funneling, Urgency

## Abstract

Enhörning’s abdominal pressure transmission theory (ET) is built on Pascal’s law of fluid pressures. A theory that rejects ET also rejects this basic physical law and cannot be considered scientifically sound. The integral theory (IT) of female stress urinary incontinence rejects ET. This issue is discussed from the viewpoint of the urethral hanging theory of female stress urinary incontinence (UHT).

## Discussion

Enhörning’s abdominal pressure transmission theory (ET) is built on Pascal’s law of fluid pressures, which states that when there is an increase or change in pressure at any point of a confined fluid entity there will be an equal increase or change at every other point in the confinement. The abdominal cavity (AC) is a confined “water bag” entity, which is caudally limited by the pubocervical fascia (PCF). Accordingly, the abdominal pressure (Pabd) is the same throughout the AC, except for a hydrostatic pressure component. This is true for both continent and incontinent women, both at rest and during stress. The bladder and the proximal urethra are situated on the PCF, and are thus inside the AC, and within the pressure equalization zone.

Petros and Ulmsten have rejected ET, and consequently, have also rejected Pascal’s basic physical law. Instead, they hypothesized that pressure transmission “is most likely an index of a changed intraurethral area” where the posterior pubourethral ligaments (PUL) constitute a fulcrum against which the proximal urethra is kinked, stretched, and narrowed by three bidirectional striated pelvic muscles [1]. In other words, urethral closure is due to a musculoelastic mechanism and not to pressure transmission.

In a recently published article [2], Petros criticizes the UHT [3–5] and states that I have misquoted his and Ulmsten’s 1995 experiment, which “invalidates all pressure transmission theories” [6]. In this experiment, five women with genuine stress urinary incontinence (SUI) were studied during a midurethral sling operation performed under local anesthesia and sedation. Pressures were measured with the anterior vaginal wall closed (intact) and opened (not intact), using microtransducers inside the urethra at the midurethral high-pressure zone, and in an equivalent position just outside the urethra wall.

The experiment indicated that during stress (coughing) with an intact anterior vaginal wall, the maximum urethral pressure during stress (sMUP; s=stress) was much higher than the pressure just outside the urethral wall (Pabd), and Petros states that “the only possible explanation for this was a muscular reflex which actively closed the urethra.” I assume that Petros here refers to the musculoelastic mechanism described in the text above.

My comment: ET and the UHT consider otherwise. To cough, a woman must produce an acutely high Pabd. Such a pressure increase is created by simultaneously contracting all the muscles surrounding the AC, including the diaphragm and the pelvic floor muscles (PFM). The urogenital rhabdosphincter complex (RS), which includes the sphincter urethrae, the compressor urethrae and the urethrovaginal sphincter muscles has the same somatic innervation as the PFM (S2-S4), and contracts synchronously with them. These synchronized contractions can be defined as a guarding reflex (GR), which actively contributes to keep the urethra closed. The noradrenergic and serotonergic signals from the pontine micturition center, which stimulate the sacral micturition center (S2-S4), to contract the RS (Onuf's nucleus/pudendus nerve), likewise stimulate the thoraco-lumbal sympathetic nucleus (Th10-L2/hypogastric nerve) to contract the urethral circular smooth muscle (α1-adrenoceptors). The contribution of the pudendal nerve to the innervation of the levator ani muscles is disputed. However, this does not exclude a simultaneous 100% transmission of the increase in the abdominal pressure (ΔPabd). sMUP = MUP + GR + ΔPabd > Pabd; where MUP is the maximum urethral pressure at rest, generated by intrinsic smooth muscles, the resting tonus of the RS, the vascular plexus, collagen and elastic fibers, the transmitted Pabd at rest, and the high resting tonus of the PFM which close the urogenital hiatus and lift the PCF, pressing the posterior urethral wall against its anterior wall. These structures, which generate the urethral closure at rest, recoil to achieve closure after cessation of urine flow. The posterior PUL extend into the anteromedial part of the PFM (the pubococcygeus muscles) and are concomitantly elevated, thereby sustaining the correct spatial relationship between the proximal urethra and the bladder neck. 

The 1995 experiment also showed that, with a not-intact anterior vaginal wall, the sMUP when coughing increased by up to 170%; “yet all patients leaked large amounts of urine”. When the vagina was reattached after placement of the sling (intact vaginal wall), all patients were dry on coughing. Petros argues, “If the pressure transmission theory were correct, the very high pressure rise (170%) would have closed the urethra and there would be no leakage”.

**Fig. 1:**
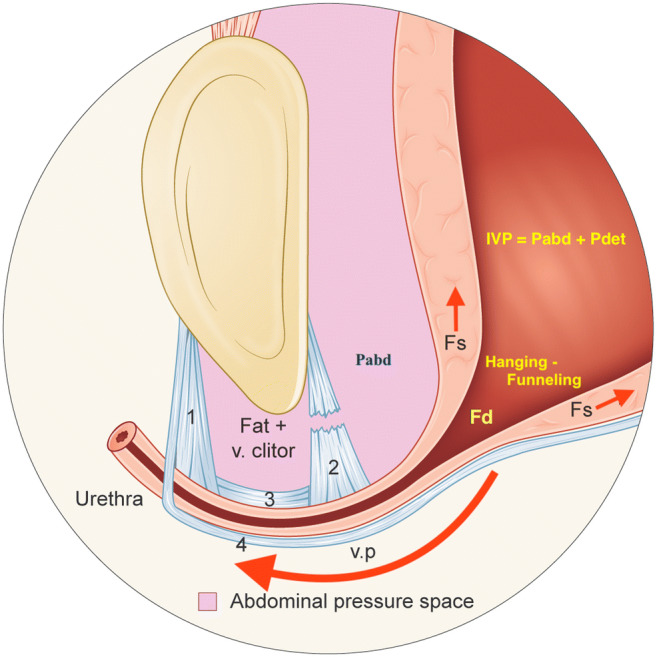
Illustration of hypermobile stress urinary incontinence during a Valsalva maneuver. In the illustrated case the Pabd is less than the abdominal leak point pressure (aLPP) and thus there is hanging/"forced funneling" without urine leakage. The maximum urethral pressure during stress (sMUP) resists the distending force (Fd) but the enforced distension of the proximal urethra may provoke urgency and frequency symptoms [5].*1* right anterior pubourethral ligament which attaches to the pubocervical fascia (PCF), *2* right posterior pubourethral ligament which attaches to the PCF, *3* right intermediate pubourethral ligament which attaches to the PCF, *4* pubocervical fascia (PCF), *Fd* outflow distending force, *Fs pulling/* shearing force, *v. clitor* vena clitoridis, *v.p.* vaginal point (which corresponds to the attachment point of the posterior pubourethral ligaments (PUL) to the pubocervical fascia on each side of the urethra), *IVP* intravesical pressure, *Pabd* abdominal pressure, *Pdet* detrusor pressure. The illustration can alternatively be interpreted to demonstrate a urethra with minimal mobility (“fixed urethra”), exhibiting hanging/“forced funneling”, even at rest [5]

My comment: this logic is incorrect [7] . The proximal urethra is inside the AC and its pressure during stress (sMUP) rises synchronously with the Pabd. There is a zero-sum situation, even though the pressures are higher. If a woman is continent at rest, she is also continent under stress. In SUI, however, the situation is different. When the Pabd is high enough to force the proximal urethra down to a hanging position on a less mobile bladder neck, the meatus internus (m.i.) is opened by a pulling/shearing force (Fs) and an outflow distending force (Fd). This counteracts the high sMUP (sMUP – Fs – Fd < Pdet + Pabd), where Pdet is the detrusor pressure (Fig.1). The size of the increase in sMUP before leakage depends on how much the Pabd has to rise before the proximal urethra is pressed down to a hanging position. This pressure is called the abdominal leak point pressure (aLPP), which can be low or high regardless of a low or high MUP or hyper−/hypomobile SUI. These inconsistencies are explained by the fact that there is no absolute covariation between defective support tissues extrinsic to the urethra and deficient urethral intrinsic tissues, or with defective support of the urethra in relation to the bladder neck. The aLPP is predominantly related to the compliance of the proximal urethral support in relation to the compliance of the bladder neck support. A more compliant urethral support prompts the proximal urethra to descend to a hanging position on a less mobile bladder neck. The mobility of the proximal urethra in relation to the bladder neck determines whether SUI occurs; the descent of the proximal urethra is unimportant if the bladder neck descends correspondingly. The placement of the sling cures SUI by preventing the descent of the urethra into ahanging position and not by restoring a midurethral fulcrum.

Thus, the sMUP, low or high, has no impact on opening of the m.i.; however, it is important for the secondary closure mechanism. Conversely, urethral funneling has a fundamental impact. If the radius of the m.i. increases from 0.5 to 5 mm, the Fd increases 100-fold (Pascal’s formula F=P*area). The m.i. is the primary closure mechanism, and the midurethral high-pressure zone is the secondary closure mechanism. This secondary closure mechanism is important for voluntary interruption of flow, emptying the urethra after micturition and obstructing retrograde flow during activities such as swimming.

According to the UHT, the proximal urethra is closed by recoiling [5], pressure transmission, and a guarding reflex. The normal posterior PUL sustain the correct spatial relationship between the proximal urethra and the bladder neck, preventing the urethra from descending to a hanging position. A closed m.i. is a perfect seal that cannot be pushed open; it must be pulled open.This is in accordance with the law of elastic collision, which states that a molecule bouncing against the bladder wall generates a force perpendicular to it. Therefore, a urethra that descends without reaching a hanging position on a less mobile bladder neck will not generate a pulling force. There is no need for an external musculoelastic mechanism where three bidirectional striated pelvic muscles close the proximal urethra using the vulnerable posterior PUL as a fulcrum. The bladder–urethra complex constitutes one organ with inherent internal mechanisms for closing, opening, relaxing, and contracting.

During normal micturition, the urethral circular smooth muscle, the RS, and the PFM relax whereby the pelvic floor/PCF descend to a lower level. The posterior urethro-vesical angle (PUVA) widens when the posterior PUL descend together with the PFM. Coordinated with the bladder contraction, the m.i. is pulled open when the curved, conjoined, inner longitudinal smooth muscles of the bladder and urethra, which are innervated by parasympathetic nerves, contract, shorten and straighten. The described increase in the PUVA during micturition (“relaxed” PUL) is analogous to what happens in SUI (defected PUL) when the proximal urethra is “unintentionally” pressed down to a hanging position. This possibly explains why normal continent women can fascilitate urination by straining. Without such a hanging mechanism that additionally funnels the proximal urethra, straining/pushing would only resultin a “zero-sum situation” described in the text above, without any effect on bladder emptying.

Realizing that urethral hanging occurs, is the key to understanding the pathophysiology of SUI and MUI (mixed urinary incontinence). Urethral hanging with enforced distension of the proximal urethra may provoke urgency and frequency symptoms [5, 7, 8]. To cure SUI and MUI the urethra must be prevented from hanging on a less mobile bladder neck. This includes, in the case of hypermobile SUI, a tension-free suburethral support at the vaginal point (v.p.), and, in the case of hypomobile SUI, a lifting support where the urethra at the v.p., is elevated above its resting position (Fig. 1) [5]. To create a lift without the risk of obstruction, the “TVT technique” can be employed to insert one tuned tape into the paraurethral tissue on each side of the v.p., or alternatively to elevate the proximal urethra by broadly folding the PCF at the v.p. and then supporting the plicated fascia with a tension-free suburethral tape (TVT). A TVT placed starting at 1 cm from the bladder neck implies that the center of the tape is positioned at the v.p..

The IT rejects Pascal’s law of fluid pressures and, consequently, it cannot be considered scientifically sound. The UHT is a biomechanical model —built on a new idea, the works of others, and the laws of physics — that explains the pathophysiology of SUI and MUI and accordingly how to repair defective anatomy.
